# Deregulation of subcellular biometal homeostasis through loss of the metal transporter, Zip7, in a childhood neurodegenerative disorder

**DOI:** 10.1186/2051-5960-2-25

**Published:** 2014-02-28

**Authors:** Alexandra Grubman, Grace E Lidgerwood, Clare Duncan, Laura Bica, Jiang-Li Tan, Sarah J Parker, Aphrodite Caragounis, Jodi Meyerowitz, Irene Volitakis, Diane Moujalled, Jeffrey R Liddell, James L Hickey, Malcolm Horne, Shoshanah Longmuir, Jari Koistinaho, Paul S Donnelly, Peter J Crouch, Imke Tammen, Anthony R White, Katja M Kanninen

**Affiliations:** 1 Department of Pathology, The University of Melbourne, Parkville, VIC 3010, Australia; 2 AI Virtanen Institute for Molecular Sciences, University of Eastern Finland, Kuopio FI-70211, Finland; 3 Florey Institute of Neuroscience and Mental Health, The University of Melbourne, Parkville, VIC 3010, Australia; 4 School of Chemistry and Bio21 Molecular Science and Biotechnology Institute, The University of Melbourne, Parkville, VIC 3010, Australia; 5 ReproGen, Faculty of Veterinary Science, The University of Sydney, Camden, NSW 2570, Australia

**Keywords:** Biometal homeostasis, Neurodegeneration, Zip7, Neuronal ceroid lipofuscinoses, CLN6

## Abstract

**Background:**

Aberrant biometal metabolism is a key feature of neurodegenerative disorders including Alzheimer’s and Parkinson’s diseases. Metal modulating compounds are promising therapeutics for neurodegeneration, but their mechanism of action remains poorly understood. Neuronal ceroid lipofuscinoses (NCLs), caused by mutations in *CLN* genes, are fatal childhood neurodegenerative lysosomal storage diseases without a cure. We previously showed biometal accumulation in ovine and murine models of the CLN6 variant NCL, but the mechanism is unknown. This study extended the concept that alteration of biometal functions is involved in pathology in these disorders, and investigated molecular mechanisms underlying impaired biometal trafficking in CLN6 disease.

**Results:**

We observed significant region-specific biometal accumulation and deregulation of metal trafficking pathways prior to disease onset in *CLN6* affected sheep. Substantial progressive loss of the ER/Golgi-resident Zn transporter, Zip7, which colocalized with the disease-associated protein, CLN6, may contribute to the subcellular deregulation of biometal homeostasis in NCLs. Importantly, the metal-complex, Zn^II^(atsm), induced Zip7 upregulation, promoted Zn redistribution and restored Zn-dependent functions in primary mouse *Cln6* deficient neurons and astrocytes.

**Conclusions:**

This study demonstrates the central role of the metal transporter, Zip7, in the aberrant biometal metabolism of CLN6 variants of NCL and further highlights the key contribution of deregulated biometal trafficking to the pathology of neurodegenerative diseases. Importantly, our results suggest that Zn^II^(atsm) may be a candidate for therapeutic trials for NCLs.

## Introduction

Biometals including Zn and Cu are immensely important for brain function. When homeostatic control fails in aging or disease, biometal mislocalization or altered homeostasis can drive pathological changes as observed in patients suffering from neurodegenerative diseases [1], as the brain is especially vulnerable to metal-induced oxidative stress. Importantly, even subtle changes to biometal concentrations have been associated with significant neuronal pathology [2].

Neuronal ceroid lipofuscinoses (NCLs), commonly known as Batten diseases, are a genetically heterogeneous group of lysosomal storage diseases (LSDs) [3], characterized by storage of lipofuscin material in lysosome-derived fluorescent storage bodies [4,5] and neuronal demise leading to progressive loss of vision, motor dysfunction and premature death [6]. Oxidative stress and neuroinflammation phenotypes in NCLs are reminiscent of other forms of neurodegeneration. Alternative mutations in the metal transporter ATP13a2 have been implicated in an NCL and early onset Parkinsonism, suggesting a common pathway [7-11], however the functions of the NCL proteins and underlying disease processes are not well understood. A variant late infantile and an adult onset NCL are caused by mutations in *CLN6,* which encodes a highly conserved transmembrane endoplasmic reticulum (ER) protein of unknown function [12,13].

We previously demonstrated region-specific alterations to biometal homeostasis in ovine CLN6 disease that correlated with the development of neurodegeneration [14]. Moreover, biometal accumulation was associated with region-specific loss of *Cln6* mRNA in presymptomatic *Cln6* affected mice [15]. However, the mechanisms responsible for these changes remain unknown. Here we show that altered biometal trafficking pathways involve loss of ER-co-localized transmembrane proteins, CLN6 and the metal transporter Zip7, triggering subcellular metal accumulation in presymptomatic CLN6 disease. Moreover, correction of impaired metal-dependent functions in *Cln6* cells is achieved via up-regulation of Zip7 by a cell permeable metal complex, Zn^II^(atsm). These studies demonstrate the potential of biometal modulation for the development of therapeutics for CLN6 disease.

## Materials and methods

### Sheep

The *CLN6* Merino and South Hampshire research flocks were maintained under standard pasture conditions on University research farms and genotyped as described [16]. As previously reported, Merino *CLN6* sheep are phenotypically normal until the age of 8–12 months [17]. From approximately 8 months, the affected sheep exhibit mild behavioural changes and visual impairment, which progress throughout disease course, resulting in premature death between 19 to 27 months of age [17]. Similar to the South Hampshire *CLN6* model, the affected Merino sheep brains do not develop normally to maturity and begin to atrophy from approximately 6 months of age. The genotype of Merino sheep was determined by genotyping for the disease causing c.184C > T mutation in the *CLN6* gene [18]. Homozygous normal animals were used as controls. *CLN6* South Hampshire sheep were used as an additional model of CLN6 disease. For comparison to affected *CLN6* South Hampshire sheep, we used unaffected homozygous controls. As an additional control group, we used unaffected heterozygous *CLN5* Borderdale sheep, as previously described [19]. All animal procedures were carried out according to NIH guidelines, the NSW Animal Research Act (1985), the New Zealand Animal Welfare Act (1999), and the Australian Code of Practice for the Care and Use of Animals for Scientific Purposes 7th Edition (NHMRC 2004). Brain and peripheral samples were collected from 3, 7 and 14 month-old control and *CLN6* affected Merino sheep, 12–14 month-old control and *CLN6* affected South Hampshire sheep and unaffected *CLN5* heterozygote Borderdale sheep. At post mortem, brains were dissected into the following regions: occipital lobe, parietal lobe, frontal lobe, thalamus, cerebellum and brain stem, and immediately frozen. Liver and muscle (rectus femoris) tissue was also collected for analysis.

### Mice

Animal handling and experimentation were performed in accordance with national and institutional guidelines (University of Melbourne AEC no. 1112024). The genotypes of affected *Cln6* mice [20] (B6.Cg-*Cln6nclf*/J, The Jackson Laboratory) were determined as previously described [15]. Mice were euthanized by CO_2_ asphyxiation or cervical dislocation, as appropriate.

### Cell culture

Primary cortical neuronal cultures were established from embryonic day 14 (E14) mice, and primary astrocytes were harvested from neonatal mice as previously described [21]. For primary cortical cultures, E14 mouse cortices were removed, dissected free of meninges and dissociated in 0.025% trypsin. Viable dissociated cells were suspended in Minimum Eagle’s Medium supplemented with 10% fetal bovine serum (FBS), 5% horse serum, 1% glutamine, and 10 μg/mL gentamicin, plated into Poly-D-lysine coated culture plates, and incubated at 37°C in 5% CO_2_. Growth medium was replaced with Neurobasal growth medium supplemented with B27, glutamine and gentamicin the following day. 3–4 days after plating, half of the media was replaced with fresh Neurobasal media. Cells were used for experiments 6 days after plating.

For astrocyte culture, newborn mice were decapitated, the brains removed and placed into ice-cold preparation buffer (containing 68 mM NaCl, 2.7 mM KCl, 110 μM KH_2_PO_4_, 84.5 μM Na_2_HPO_4_, 29 mM sucrose, 2.8 mM glucose, 20U/mL penicillin and 34.4pM streptomycin). Brains were diced, then sequentially passed through 250 μm and 135 μm gauze and centrifuged at 500×g for 5 min. Cell pellets were resuspended in growth medium (high glucose DMEM containing 10% FCS, 20U/mL penicillin and 34.4pM streptomycin) and plated into 6 well plates at 1.5×10^6^ cells/well. Cells were maintained at 37°C with 10% CO_2_. Growth medium was replaced every 7 days, and experiments were performed after 16 days *in vitro.*

Zn^II^(atsm) was added to cells cultured in Neurobasal medium for neurons or high glucose DMEM (Life Technologies) containing 10% FCS, 20 U/mL penicillin and 34.3pM streptomycin for astrocytes. High content screening analysis was used to visualize and quantitate neurite characteristics of primary neurons, as described in supplementary methods.

### Metal analyses

The metal contents in sheep occipital, parietal and frontal lobes, cerebellum, thalamus, brainstem, liver and muscle were measured using inductively coupled plasma mass-spectrometry (ICP-MS) as before [14].

### Cathepsin D assay

Cathepsin D activity was measured using a Fluorometric cathepsin D activity assay kit (Abcam, Cambridge, MA).

### Alkaline phosphatase (ALP) assay

ALP activity assays were performed on primary mouse astrocytes, as described [22], *p*-nitrophenol release being measured at 405 nm over 30 min using shrimp ALP (Sigma-Aldrich, Castle Hill, NSW, Australia) as a standard.

### FluoZin-3 fluorescence

Mouse neurons were pre-treated with 5 μM FluoZin-3 (Life Technologies) for 30 min, followed by a 30 min washout. Fluorescence was measured with an Enspire plate reader (PerkinElmer, Glen Waverley, Victoria, Australia) in well scan mode at excitation and emission wavelengths of 494 and 516 nm, respectively.

### High content screening analysis

Primary cortical mouse neurons plated in clear bottom Costar 96 well plates (Corning, Tewksbury, MA) were treated with Zn^II^(atsm) [23] for 1 h, at the concentrations indicated. Cells were fixed for 15 min in 4% PFA in phosphate buffered saline (PBS), permeabilised for 10 min at -20°C with ice-cold 100% methanol, and blocked with 5% FCS and 0.03% Triton-X100 in PBS. Zip7 complexes were detected with rabbit α-Zip7 (1:500; ProteinTech, Chicago, IL) in dilution buffer (1% BSA, 0.03% Triton-X100 in PBS) and goat-α-rabbit IgG AlexaFluor 647 (1:500, Life Technologies). Cell nuclei were stained with DAPI (1.5 μM, Life Technologies). For the measurement of neurite lengths, cellular tubulin was stained with rabbit α-tubulin antibodies (1:50; Cell Signaling, Arundel, Queensland, Australia). Staining in cells was viewed with a *Cellomics* ArrayScan VTI HCS Reader (Thermo Scientific, Scoresby, Victoria, Australia). The *Cellomics* ArrayScan VTI HCS Reader is a high throughput microscopy-based screening analysis platform, which provides highly reproducible quantitative and qualitative data that can be obtained from cell populations in 96 well plate format. Due to rapid data acquisition, the ability to measure 1000s of cells per treatment condition and lack of experimenter bias, the *Cellomics* technology is now well accepted for routine use in neurite outgrowth assays, as evidenced by a number of highly cited publications [24-26]. At least 1,000 cells or 20 fields per well were captured with the 20× objective. Data were analyzed with vHCS 116 Discovery Toolbox software (Thermo Scientific), using the compartmental analysis or neuronal profiling bioapplications, as appropriate (Additional file 1). For analysis of Zip7 staining intensity, the nucleus was identified by DAPI staining using the vHCS 116 Discovery Toolbox software. The perinuclear region of each cell was designated as beginning 2 μm from the perimeter of the nucleus, and extending for a 2 μm radius around the nucleus. The average pixel intensity of this region was calculated and plotted for at least 1000 cells per well, performed in triplicate wells. The experiment was repeated 4 times with similar results.

### Immunofluorescent staining

Immunofluorescent staining was performed as previously described [27]. Briefly, primary cortical mouse neurons were seeded onto coverslips in 24 well plates, treated with Zn^II^(atsm) for 4 h, fixed in 4% PFA, permeabilised in 0.1% Triton-X100 in PBS, and blocked in 5% FCS in PBS. Zip7 was detected using polyclonal rabbit-α-Zip7 antibodies (1:500), and goat-α-rabbit AlexaFluor 488 (1:500; Life Technologies). Cell nuclei were visualized with DAPI. Coverslips were mounted onto microscope slides with fluorescence mounting media (DAKO, Campbellfield, Victoria, Australia) and visualized with a Leica DMIRB fluorescence microscope. For colocalization studies, primary mouse cortical neurons were reacted with rabbit primary anti-CLN6 and goat primary anti-Zip7 antibodies. Anti-goat AlexaFluor-488 and anti-rabbit AlexaFluor-568 dye labeled secondary antibodies were used to reveal Zip7 and CLN6 expression, respectively. ER localization was determined by staining with the rabbit polyclonal antibodies to the ER marker, calnexin (1:100; Abcam) and anti-rabbit AlexaFluor-568 dye labeled secondary antibodies. Fluorescence was visualized by confocal microscopy using the Zeiss Meta confocal scanning laser microscope with a magnification of 40x.

### qRT-PCR

RNA was prepared from 10^6^ primary mouse neuronal cells using the MagMax Total RNA isolation kit (Life Technologies). RNA (200 ng) was reverse transcribed using the High Capacity cDNA kit (Life Technologies). TaqMan gene expression assays for *MT1A* and *TUBA8* were purchased from Life Technologies (Mm00496660_g1 and Mm00833707_mH, respectively) and qRT-PCR was performed as previously described [15]. Delta Ct method was used for normalization of expression relative to β-tubulin.

### siRNA transfection

Mouse NIH 3T3 cells (6×10^5^/mm^2^) were cultured for 24 h in DMEM supplemented with 10% FBS, 1% 20 U/mL penicillin, 34.4pM streptomycin, 1% L-Glutamine, 1% HEPES and 1% non essential amino acids. Transfection was achieved with DharmaFECT transfection reagent 4 (Thermo Scientific) and 100 nM Stealth Select RNAi siRNA specific to Zip7 (oligo ID MSS205001) or Negative control High GC siRNA (both from Life Technologies) according to manufacturer specifications. siRNA-transfection reagent complexes were incubated for 20 min in DMEM prior to dropwise addition to cells. Cell media was changed after 24 h to DMEM containing 10% FBS. 72 h after transfection, cells were harvested for Western blotting by scraping and centrifugation (18,000×g, 5 min).

### Western blotting

Cell lysates and tissues homogenized with a Dounce tissue grinder were extracted with Phosphosafe (Merck, Kilsyth, Victoria, Australia) containing a protease inhibitor cocktail (Roche, Castle Hill, NSW, Australia) and DNAse (Roche). The protein concentrations of supernatants after centrifugation (12,000×g, 5 min, 4°C) were measured with the BCA assay kit (Pierce) according to manufacturer’s instructions. Equal protein amounts were separated on 12% SDS-PAGE Tris-glycine or 4-12% Bis-Tris gels (Life Technologies), as appropriate. Proteins were transferred to PVDF membranes and blocked with 4% skim milk solution in PBS-Tween. Membranes were probed overnight with primary antibodies diluted in 4% skim milk solution in PBS-Tween. Unless stated otherwise, primary antibodies were raised in rabbits and were diluted 1:1000. The antibodies used in this study were directed against: CLN6 (kindly provided by Dr. Sara Mole, University College London), mouse Zip3 (Abnova), Zip7 (1:2000, Proteintech), Zip8 (1:1200, Proteintech), Zip14 (Novus), ZnT1 (Sigma), ZnT3 (Proteintech), ZnT6 (1:1200, Proteintech), ZnT7 (Proteintech), mouse α-synuclein (1:2000, kindly provided by Professor Malcolm Horne, The Florey Institute of Neurosciences), V-ATPase (GenScript), as well as the phosphorylated form of GSK-3 (Cell Signaling Technologies). The horseradish peroxidase-conjugated anti-mouse or anti-rabbit secondary antibodies (Cell Signaling Technologies) were used at a dilution of 1:5000. Membranes were developed by chemiluminescence (Amersham ECL Advance Western blotting detection kit) and imaged on a Fujifilm LAS3000 Imager (Berthold). Western blots were subjected to densitometry analysis using ImageJ software.

Target band intensities were compared to control bands (ImageJ, Bethesda, MD) on blots probed with antibodies against GAPDH, β-tubulin, total Akt or total ERK used as controls to normalize protein concentrations in CLN6 affected and control animals.

### Statistical analyses

Differences in metal content, gene expression, protein concentrations, ALP activities and neurite characteristics were determined using unpaired Student’s *t-*tests. *p* values below 0.05 were considered significant. Data are expressed as means ± SEM and are representative of a minimum of three independent experiments. Where sheep data are presented, N = three or four animals per group. Pearson correlation coefficients were used to determine the strength of correlation between Zn, P-GSK, metallothionein, CLN6 and Zip7 levels in individual sheep.

## Results

### Impaired lysosomal function and neurodegeneration-associated proteinopathy in NCLs

**Figure 1:**
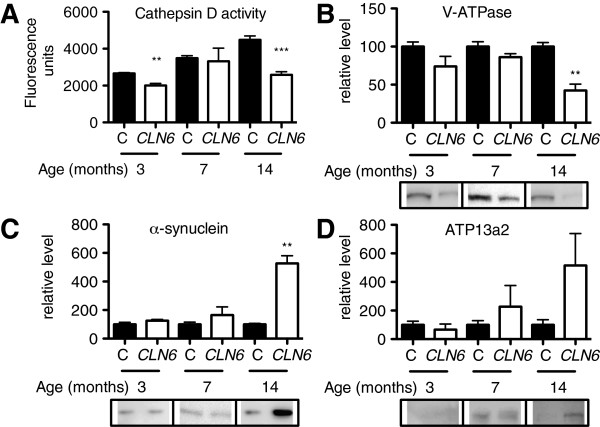
**Lysosomal dysfunction and increased α-synuclein concentrations in *CLN6 *sheep. (A)** Cathepsin D activity was measured in homogenates (1 μg) isolated from the occipital lobe of 3, 7 and 14 month old control or *CLN6* affected Merino sheep (N = 3 per group) using a fluorometric Cathepsin D activity assay. **(B-D)** Densitometry and representative immunoblots of homogenates (5–40 μg) isolated from the occipital lobe of 3, 7 and 14 month old control or *CLN6* affected sheep probed with antibodies directed against V-ATPase **(B)**, α-synuclein **(C)** or ATP13a2 **(D)**. GAPDH was used as a loading control. Quantification was performed in ImageJ and concentrations are expressed relative to those in control sheep at each age. Data are mean + SEM. **p < 0.01, ***p < 0.001 by Student’s *t* test. **C**, control.

Lysosomal dysfunction, prior to the onset of clinical signs in *CLN6* affected Merino sheep, which occurs at 8–12 months [17], was demonstrated by reduced cathepsin D activity from 3 months of age (Figure 1A). An age-dependent reduction of subunit B2 of V-ATPase was also observed in the occipital lobe, the site of initial neuropathological changes of South Hampshire *CLN6* sheep (Figure 1B) [28], suggestive of defects in lysosomal functionality [29]. Due to the close relationship between NCLs and Parkinson’s disease, we examined the levels of Parkinson’s disease proteins, α-synuclein, and the metal transporter, ATP13a2. Significantly increased concentrations of the high molecular weight form of α-synuclein [30] and a trend toward increased ATP13a2 levels (*p =* 0.1782) were also observed after onset of neurological disease (Figure 1C-D), providing further support for a relationship between Parkinson’s and NCL diseases.

### Biometals accumulate in preclinical *CLN6* sheep

We previously reported accumulation of Zn, Mn, Co and Cu in disease-affected brain regions of *CLN6* Merino and South Hampshire sheep after the onset of clinical signs [14]. To investigate biometal changes in early NCL pathology, we determined metal concentrations in 3 and 7 month old normal and affected Merino sheep (Table 1). Zn concentrations were higher in the parietal lobe and muscle of affected sheep at 7 months of age, but lower in the liver of 3 month-old and plasma of 7 month-old affected sheep. Liver Mn concentrations were substantially reduced, and plasma Co reduced at 3 and 7 months of age (Additional file 2). Cu concentrations in the occipital and frontal lobes were higher in affected animals at both ages while those in the parietal lobe, brainstem and cerebellum where higher than control values at 3 months but lower at 7 months. Increased Cu concentrations were also observed in the liver of 3 and 7 month old *CLN6* sheep. Together, these data indicate that disturbances to biometal homeostasis may precede detectable clinical signs in CLN6 disease. Moreover, biometal alterations not only occur in the central nervous system (CNS), but also peripherally in NCL affected animals.

**Table 1:** **Increased biometal concentrations in the brain of preclinical *CLN6 *** **Merino sheep**

		**Zn**^**a**^		**Cu**^**a**^	
	**Age**	**Control**	***CLN6***	**Control**	***CLN6***
Frontal	**3**	11.1 ± 0.2	11.1 ± 0.7	2.0 ± 0.2	2.3 ± 0.2*
	**7**	11.4 ± 0.2	12.1 ± 1.4	2.2 ± 0.3	2.7 ± 0.6
Occipital	**3**	11.2 ± 0.4	11.2 ± 1.2	2.2 ± 0.5	2.8 ± 0.3*
	**7**	13.1 ± 0.5	13.9 ± 1.9	2.5 ± 0.2	3.4 ± 0.9*
Parietal	**3**	10.7 ± 0.9	11.0 ± 0.4	1.8 ± 0.3	2.5 ± 0.1^
	**7**	12.9 ± 0.6	13.6 ± 0.4*	2.5 ± 0.4	2.1 ± 0.3
Thalamus	**3**	10.7 ± 1.3	10.5 ± 0.6	2.1 ± 0.4	1.8 ± 0.1
	**7**	10.6 ± 1.0	11.6 ± 1.3	2.7 ± 1.2	2.7 ± 1.3
Cerebellum	**3**	12.2 ± 1.1	12.5 ± 1.3	2.8 ± 0.4	3.0 ± 0.5
	**7**	11.4 ± 0.5	11.6 ± 0.5	3.4 ± 0.5	2.3 ± 0.4^
Brainstem	**3**	11.2 ± 1.0	11.7 ± 0.6	2.5 ± 0.3	2.8 ± 0.3*
	**7**	8.52 ± 0.8	9.2 ± 0.4	1.9 ± 0.2	1.5 ± 0.4
Liver	**3**	53.9 ± 18.5	36.3 ± 9.2*	20.2 ± 2.6	37.1 ± 1.6^#^
	**7**	28.1 ± 5.3	29.5 ± 3.0	40.4 ± 7.7	58.2 ± 6.5^#^
Muscle	**3**	22.0 ± 4.5	21.5 ± 1.9	0.6 ± 0.4	0.5 ± 0.2
	**7**	19.9 ± 3.0	27.3 ± 4.1^	0.2 ± 0.1	0.3 ± 0.2
Plasma	**3**	11.7 ± 1.1	12.0 ± 2.1	13.6 ± 2.0	14.3 ± 2.7
	**7**	14.0 ± 1.4	10.5 ± 1.8*	12.8 ± 1.1	10.2 ± 2.2

^a^Metal concentrations in the CNS and peripheral tissues of 3 and 7 month old control and *CLN6* Merino sheep were measured using ICP-MS. The concentrations of Zn and Cu in each tissue are expressed as mean ± S.D. Values correspond to μg metal/g tissue. *p < 0.05, ^*p* < 0.01, ^#^*p* < 0.001 by Student’s *t* test.

### Region-specific alterations to biometal trafficking pathways are associated with loss of CLN6

**Figure 2:**
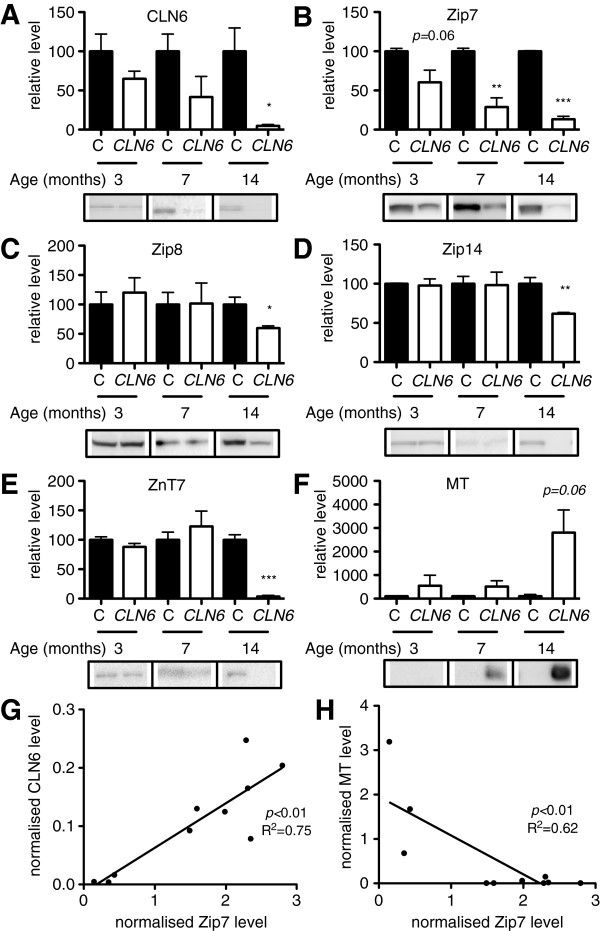
**Altered biometal trafficking pathways are associated with loss of CLN6 in sheep. (A-F)** Densitometry and representative immunoblots of homogenates (5–40 μg) isolated from the occipital lobe of 3, 7 and 14 month old control or *CLN6* affected sheep (N = 3 per group) probed with antibodies directed against CLN6 or a range of metal transporters or metal binding proteins. GAPDH, β-tubulin, total Akt or total ERK, as appropriate, were used as loading controls. Quantification was performed in ImageJ and levels are expressed relative to those in control sheep at each age. Data are mean + SEM. *p < 0.05, **p < 0.01, ***p < 0.001 by Student’s *t* test. C, control. **(G-H)** Normalized Zip7 protein levels in Merino sheep occipital lobe were plotted against normalized levels of CLN6 **(G)** or MT **(H)** to determine correlations between these proteins. Linear regression analysis was performed in GraphPad Prism.

Western blotting revealed an age related decline in the relative amount of CLN6 in the affected occipital lobe, resulting in very low levels at 14 months (Figure 2A). We assessed the expression levels of the Cu transporters, CTR1, ATP7A, ATP7B, but observed no differences in expression of these proteins between control and *CLN6* Merino sheep (unpublished data). We next focused on Zip and ZnT metal transporter proteins, initially identified for Zn transport and reported to transport different subsets of the biometals altered in *CLN6* affected sheep [31-35]. The ER/Golgi-resident transporter, Zip7, was progressively lost in the affected occipital lobe in an age-dependent manner from 3 months, reaching statistical significance from 7 months (Figure 2B, full blot of Zip7 shown in Additional file 3). Significantly reduced expression of Zip8, Zip14 and ZnT7 in affected Merino sheep was apparent by 14 months of age (Figure 2C-E), whereas ZnT1, ZnT3 and ZnT6 concentrations were relatively unchanged (Additional file 4). Similar alterations to the concentrations of metal trafficking proteins were also observed in 12 month-old *CLN6* affected South Hampshire sheep, except that ZnT6 expression was reduced, while ZnT7 expression was unaffected in this model (Additional file 5). The Zn and Cu sequestering protein, metallothionein (MT), was upregulated ~40 fold in Merino *CLN6* sheep at 14 months of age (Figure 2F) consistent with *CLN6* affected South Hampshire sheep, as reported [14]. Correlation analysis revealed a strong positive relationship between the levels of Zip7 and CLN6 and a strong negative correlation between Zip7 and MT levels in individual sheep (Figure 2G-H).

In addition to the occipital lobe, a significant decrease in Zip7 occurred in the frontal and parietal lobes of *CLN6* affected Merino sheep by 14 months of age (Additional file 6). Significant reductions were also observed in the cerebellum and thalamus of similarly aged *CLN6* affected South Hampshire sheep (Additional file 7B-C), indicating that reduction of Zip7 occurs throughout the brain in both breeds of sheep.

**Figure 3:**
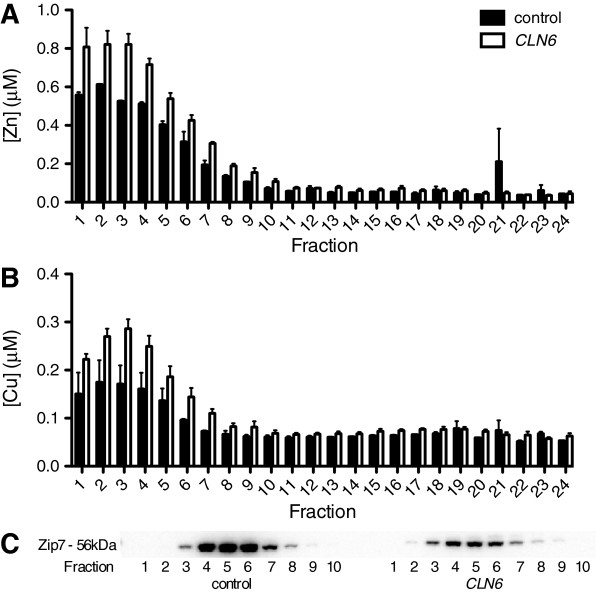
**Biometals accumulate in subcellular fractions that display Zip7 loss in presymptomatic *CLN6 *sheep occipital lobe.** Sucrose density gradient fractions from 3 month old sheep occipital lobe were analyzed for metal content by ICP-MS. Data are expressed as the mean + SEM of Zn **(A)** and Cu **(B)** concentrations in *CLN6* brains (white bars throughout) and control brains (black bars throughout) from 3 individual sheep per genotype. **(C)** Representative immunoblot of Zip7 protein present in fractions from control and *CLN6* affected sheep brains.

Brain Zn is aberrantly elevated in sucrose density gradient fractions corresponding to the ER and Golgi in *Cln6* mouse brains [15]. We therefore investigated whether deregulation of subcellular biometal homeostasis also occurred in *CLN6* sheep. To investigate early changes, before substantial changes to bulk metal levels were evident, we analyzed occipital lobes from 3-month old control and *CLN6* affected Merino sheep (Figure 3). Subcellular distribution profiles of Zn and Cu were significantly altered, with increased Zn and Cu in the lighter fractions (fractions 1–7) of *CLN6* affected brains (Figure 3A-B). Interestingly, Zip7 is lost in these fractions in *CLN6* affected brains (Figure 3C), consistent with retention of excess metals in those fractions in *CLN6* animals. We also probed fractions for CLN6, but were unable to detect CLN6 protein in any fraction (unpublished data).

### *Cln6* neurons display defects in *Cln6* transcription and perinuclear Zip7 staining

**Figure 4:**
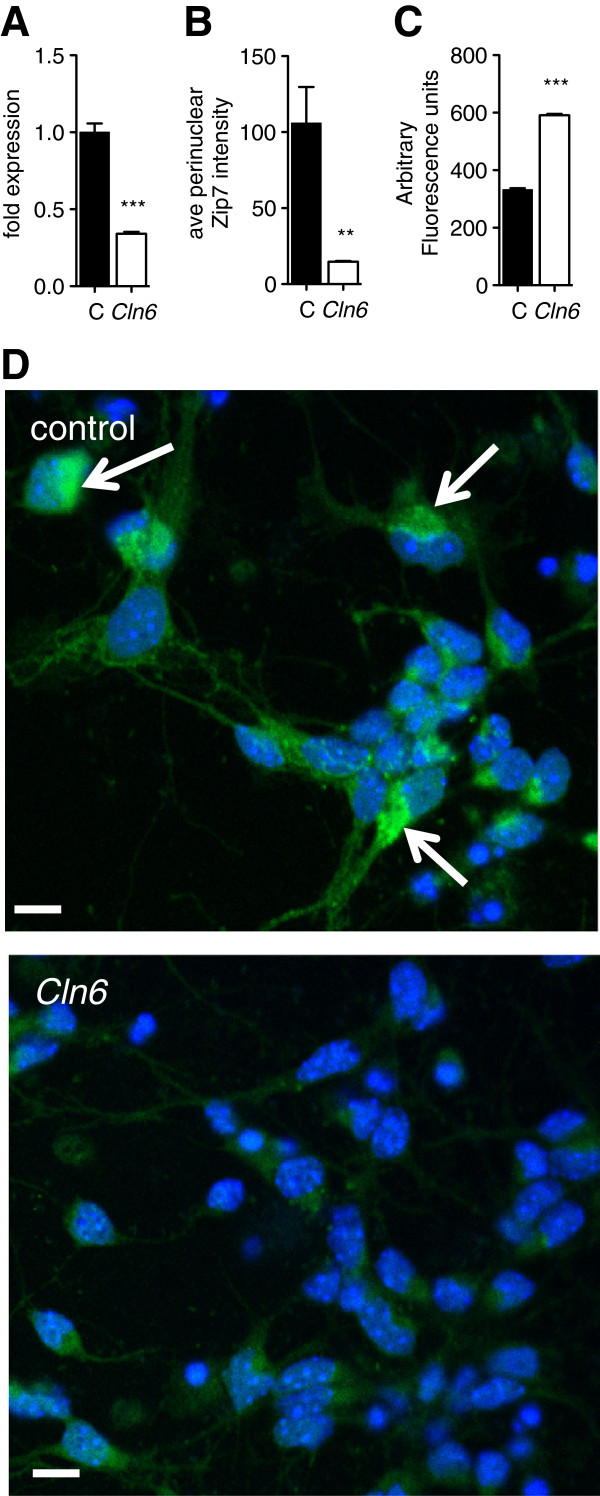
***Cln6 *cortical neurons display reduced *Cln6 *transcripts and Zip7 staining with increased labile Zn accumulation. (A)***Cln6* mRNA expression in primary murine cortical neurons was measured using qRT-PCR. Expression values were normalized to tubulin using the delta Ct method. All data are mean + SEM. **(B)** Zip7 expression was assessed by immunofluorescence. Zip7 was labeled with rabbit Zip7 antibodies and AlexaFluor conjugated anti-rabbit antibodies. Nuclei were stained with DAPI. Quantitative analysis of Zip7 perinuclear distribution was performed using the ArrayScan reader in conjunction with the compartmental analysis software on >1000 cells per genotype. **(C)** Labile Zn in control and *Cln6* cortical neurons was measured by FluoZin-3 fluorescence. **(D)** Zip7 immunofluorescence images are representative of 3 experiments performed on triplicate coverslips. Scale bars represent 10 μm.

**Figure 5:**
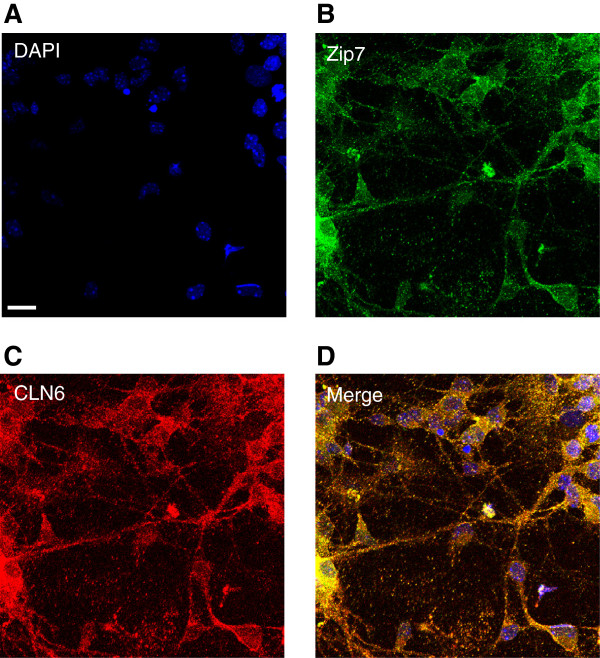
**Zip7 co-localizes with CLN6.** Primary mouse cortical neurons were reacted with rabbit primary anti-CLN6 and goat primary anti-Zip7 antibodies. Anti-goat AlexaFluor-488 and anti-rabbit AlexaFluor-568 dye labeled secondary antibodies were used to reveal Zip7 and CLN6 expression, respectively. **(A)** DAPI, **(B)** Zip7 and **(C)** CLN6 expression in primary cortical neurons was visualized by confocal microscopy using the Zeiss Meta confocal scanning laser microscope using a magnification of 40×. **(D)** Overlay images indicate colocalization in punctate structures throughout the cells. Scale bars correspond to 20 μm.

Given the challenges of *in vitro* studies in sheep, we further investigated the role of Zip7 in CLN6 disease using mice carrying a natural mutation in *Cln6*[20]. *Cln6* mRNA expression (Figure 4A) and perinuclear Zip7 staining (Figure 4B,D) were significantly reduced in neurons cultured from *Cln6* mutant mice. To investigate whether reduced Zip7 expression was associated with elevated labile Zn content, we used a cell-permeable Zn-binding fluorophore, FluoZin-3, which senses Zn that is not tightly protein-bound (K_d_ = 15 nM). Consistent with a defect in Zip7-mediated trafficking, readily exchangeable Zn pools were substantially increased in neurons harvested from *Cln6* affected mice (Figure 4C). The possibility that the ER-localized transmembrane Zip7 and CLN6 [13,36], associate was explored through confocal immunofluorescence. Cortical neurons were co-labeled with antibodies to Zip7 (Figure 5B) and CLN6 (Figure 5C). Perinuclear and punctate staining was observed throughout the cell body and neurites, suggestive of ER/Golgi localization. Importantly, a degree of colocalization between CLN6 and Zip7 was observed (Figure 5D), indicating that these proteins may associate in punctate structures throughout the cell. Co-localization of Zip7 with the ER marker, calnexin, verified that Zip7 was at least partially ER-localized (Additional file 8). These results indicate that loss of Zip7 is detectable in mice prior to birth, and provide support for an association between Zip7 and CLN6 disease.

### Delivery of bioavailable Zn restores Zn-dependent phenotypes through upregulation of Zip7

**Figure 6:**
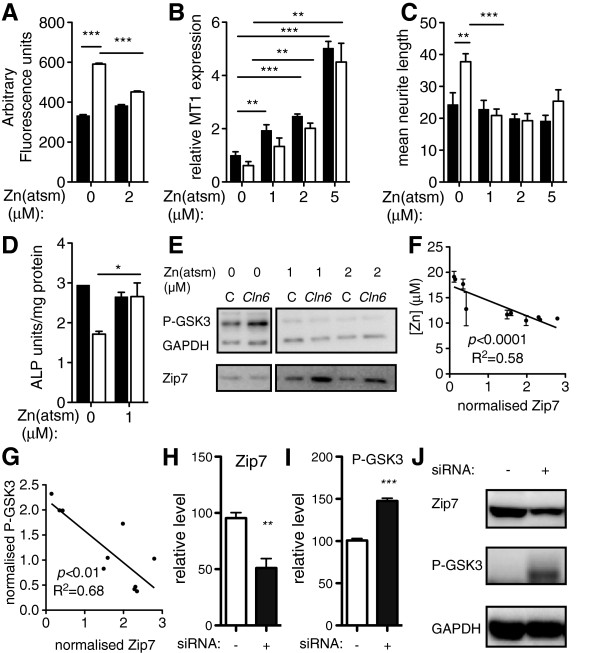
**Delivery of bioavailable Zn restores Zn-dependent phenotypes through upregulation of Zip7. (A)** Labile Zn in control (black bars throughout) and *Cln6* (white bars throughout) cortical neurons after 1 h Zn^II^(atsm) treatment was measured by FluoZin-3 fluorescence (N = 3). **(B)***MT1A* mRNA expression after 1 h Zn^II^(atsm) treatment in cortical neurons was assessed using qRT-PCR (N = 3). **(C)** Neurite length in control and *Cln6* primary cortical neurons treated for 1 h with Zn^II^(atsm) was determined by tubulin immunofluorescence. Images (>1,000 cells/well from 4 experiments) were taken using the ArrayScan High Content Platform and analysis was performed using Neuronal Profiling software (refer to Additional file 1). **(D)** ALP activity in control and *Cln6* primary mouse astrocytes was determined after 1 h Zn^II^(atsm) treatment (N = 3). **(E)** Zn^II^(atsm)-dependent regulation of P-GSK and Zip7 levels after 4 h treatment was assessed in primary mouse astrocytes by western blotting. Images are from different lanes on the same gel. **(F-G)** Normalized Zip7 protein levels in Merino sheep occipital lobe were plotted against normalized levels of Zn **(F)** and P-GSK **(G)** to determine correlations between these proteins (N = 3 sheep per genotype). Linear regression analysis was performed in GraphPad Prism. **(H-J)** Zip7 knockdown results in hyperphosphorylation of GSK3. 100nM negative control siRNA (-) or Zip7 siRNA (+) was transfected into mouse NIH 3T3 cells. Zip7 **(H)** and P-GSK3 **(I)** protein expression was determined by Western blotting and normalized to expression of GAPDH. Data are expressed as mean + SEM values. *p < 0.05, **p < 0.01, ***p < 0.001 by Student’s *t* test. **(J)** Images are representative of 3 independent experiments.

Therapeutic modulation of biometal homeostasis has demonstrated promise in the treatment of neurodegenerative disorders [37-39]. Although bulk analysis suggested overall metal accumulation in CLN6 disease, loss of the ER to cytoplasmic metal importer, Zip7, would ultimately drive metal mislocalization to the ER, as previously observed for Zn in *Cln6* mutant mouse brain [15], and may induce deficiencies of bioavailable metals elsewhere in the cell. Membrane-permeable complexes that deliver bioavailable metals may therefore bypass the Zip7 defects in *Cln6* cells. In light of this, Zn was delivered using the cell-permeable metallo-complex Zn^II^(atsm) [40]. Zn^II^(atsm) treatment significantly decreased the readily exchangeable Zn pool in primary *Cln6* neurons, as measured by FluoZin-3 fluorescence (Figure 6A). The apparent paradox of delivering Zn but observing reduced FluoZin-3 fluorescence is likely to occur via Zn^II^(atsm)-dependent upregulation of MT (Figure 6B), and therefore sequestration of excess labile Zn [41]. The results support abnormal compartmental Zn levels in *Cln6* affected cells due to Zip7 loss and the ability of Zn^II^(atsm) to at least partially rectify this by up-regulation of MT. As increased labile Zn can trigger neurite outgrowth [42], we next examined the neurite characteristics of *Cln6* neurons. Indeed, *Cln6* cells displayed longer, more extensively branched neurites, which were retracted upon 1 h treatment with up to 5 μM Zn^II^(atsm) (Figure 6C, Additional file 1). Together, these data suggest that Zn^II^(atsm) restores metal homeostasis in *Cln6* primary neurons.

Although it is neurons that degenerate in NCL disease, astrocytes are severely affected prior to disease onset in both sheep and mouse NCL models [43,44]. We therefore also examined a range of Zn-dependent activities in primary astrocytic cultures. We measured the activity of alkaline phosphatase (ALP), a Zn-dependent cytoplasmic enzyme requiring Zn loading by ZnT5 and ZnT7 for full activity [45]. Reduced ALP activity in *Cln6* astrocytes was indicative of regional Zn depletion, but was restored by 1 μM Zn^II^(atsm) treatment (Figure 6D). Elevated labile Zn can result in aberrant GSK3 phosphorylation [46], evident in *CLN6* sheep [14] and primary *Cln6* mouse astrocytes (Figure 6E). Zn^II^(atsm)-dependent rescue of hyperphosphorylated GSK-3 in *Cln6* astrocytes was associated with increased Zip7 (Figure 6E) demonstrating a link between Zn, GSK3 and Zip7 expression. Together the data suggest complex deregulation of subcellular Zn pools in *Cln6* cells - increased labile Zn in specific sub-cellular regions and reduced bioavailable Zn in alternative compartments required for enzyme functions. Importantly, our data show a strong *in vitro* protective effect of Zn^II^(atsm) on Zn mislocalization caused by *Cln6* mutation. Interestingly, Zip7 levels in individual sheep were inversely correlated to levels of P-GSK3 and Zn (Figure 6F-G), supporting functional or regulatory relationships between these proteins and cellular Zn concentrations. Consistent with a role for Zip7 in this process, siRNA knockdown of Zip7 in mouse NIH 3T3 cells resulted in hyperphosphorylation of GSK3 (Figure 6H-J). These data provide further support for a role of Zip7 in homeostatic control of a kinase that is deregulated in neurodegeneration.

## Discussion

We demonstrated that CLN6 loss is associated with significant biometal accumulation in the brains of *CLN6* affected Merino sheep prior to onset of clinical signs. We observed deregulated metal transporters belonging to the Zip and ZnT families in affected brain regions (Figure 2B-E). Zip7 was the earliest transporter altered, with loss of expression in sucrose density fractions from 3 month-old *CLN6* sheep (Figure 3) and in perinatal mouse *Cln6* cells (Figure 4B,D). Zip7 was thus the only transporter with altered expression in multiple CLN6 disease models [15], and the only ER-localized metal transporter to be altered. Thus we hypothesized that Zip7 is directly affected by loss of CLN6. Indeed, CLN6 co-localized with Zip7 in murine cortical neurons (Figure 5) and reduced Zip7 expression occurred concomitantly with elevated labile Zn in primary *Cln6* neurons (Figure 4C). This study implicates Zip7 as a potentially important contributor to NCLs.

Stringent regulation of biometal homeostasis by cellular trafficking systems is critical to prevent metal-induced toxicity. Mn overexposure causes Parkinsonism and α-synuclein accumulation [47]. The latter was observed in *CLN6* affected sheep (Figure 1B), occurs in another LSD, Gaucher’s disease [48], and was recently associated with lysosomal dysfunction in a rare genetic form of parkinsonism, Kufor-Rakeb syndrome, caused by mutations in *ATP13a2*[49]*.* Indeed, mutations, altered expression or aberrant functionality of metal transporters belonging to the ATP7, ATP13, TRMPL and ZnT families has been implicated in neurodegenerative disorders with pathological or clinical similarities to NCLs, and in the LSDs, Niemann-Pick C and Mucolipidosis [50-56]. As increased ATP13a2 expression was reported to rescue lysosomal dysfunction in Parkinson’s disease fibroblasts [57], we hypothesise that upregulation of ATP13a2 in *CLN6* affected sheep may similarly compensate to partially improve lysosomal function. Together, these studies emphasize that while the precise mechanisms underlying altered metal homeostasis in neurodegeneration are specific for each disease, neurodegenerative processes involving aberrant cellular metal trafficking are intricately linked at the molecular level.

We reported biometal elevation in the brains of 2 *CLN6* affected sheep models post-clinical onset [14], and in the brains and heart of presymptomatic *Cln6* mice [15]. Here we show significantly increased concentrations of Cu and Zn in the brains of Merino *CLN6* affected sheep prior to clinical onset. While the spatio-temporal pattern of metal concentrations is variable in tissues, this is not unexpected for several reasons. As reported for *Cln6* mice, each brain and peripheral tissue region has different localized metal levels, which can vary across disease course [15]. For instance, the stabilization in the levels of Cu in the brainstem, frontal and parietal lobes observed at 7 months (Table 1) parallels a transient improvement in lysosomal function (Figure 1A). This improvement could represent the temporary efficacy of endogenous compensatory mechanisms such as increased MT expression (Figure 2F) that ultimately fail due to presence of persistent stressors, causing animals to succumb to disease by 14 months of age when metal concentrations rise as reported [14]. Moreover, homeostasis of multiple biometals is highly inter-related, and changes in one metal can greatly affect the absorption or trafficking of others in a cell- and tissue-specific manner [58]. It is also important to recognize that, as physiological control of biometal homeostasis is so precise, even subtle focal mislocalisation of biometals can have a critical impact on cell functions, as evident in Alzheimer’s disease. Although it is now widely recognized that excess extracellular Zn and Cu can promote amyloid β aggregation [59] and studies suggest that intracellular metal levels are depleted in Alzheimer’s disease [60], these changes are rarely reflected in studies examining metal levels in bulk tissue homogenates [61]. Similarly, the subtly altered metal concentrations here, while indicative of global metal dyshomeostasis, are insufficient to completely capture the impaired subcellular biometal trafficking dynamics. This is further supported by the significant accumulation of Zn in 3 month-old brains detected in sucrose density fractions (Figure 3), but not by bulk analysis. The substantial increase in MT expression (Figure 2F), consistent with a recent report on MT overexpression in LSDs [62], is likely a response to subcellular accumulation of Zn and/or Cu in affected brain regions, but overexpressed cytoplasmic MT may not have direct access to effectively sequester mislocalized metal ions. Together these results implicate widespread loss of biometal homeostasis as an early disease feature.

Region-specific early alterations to the CLN6-colocalizing metal transporter, Zip7, in multiple CLN6 disease models indicate an important role of this metal transporter in CLN6 NCL. Visual dysfunction in CLN6 NCL may also be related to Zip7 expression changes, as Zip7 plays a role in brain and eye development in zebrafish [63]. Indeed, Zip7 loss in zebrafish caused eye defects [63], potentially a phenocopy of CLN6 disease. Moreover, loss of the *Drosophila* Zip7 homologue, Catsup, was recently implicated in amyloid-precursor like protein accumulation [64], providing additional links to Zip7 involvement in neurodegeneration. Analysis of interactions between Zip7 and different mutant forms of CLN6 may shed light on the differences in cellular Zip7 concentrations between mouse and sheep CLN6 models.

Due to high affinity interactions with proteins (as predicted by the Irwing-Williams series), the labile cellular Zn content is estimated to be in the high picomolar range [65], while ER and Golgi labile Zn concentrations are kept at a subpicomolar range [66]. Increased compartmentalized labile Zn as a result of Zip7 loss may give rise to consequential perturbations in the metabolism of other metals. For instance, excess labile Zn may displace redox-active transition metals from metalloproteins, which may participate in Fenton-type ROS-producing reactions. Additionally excess free Zn in the ER or Golgi may aberrantly bind to, and inhibit, proteins. For instance, Zn has been shown to inhibit protein tyrosine phosphatases in the ER at low picomolar concentrations [67]. Moreover, Zip7 loss may preclude kinase-dependent Zn mobilization, termed “Zn wave” signals from being propagated throughout cells [68], which may impact pleiotropic cellular signaling processes.

Further highlighting the role of Zip7 in CLN6 disease, the metal complex, Zn^II^(atsm), restored Zn-dependent functions and induced Zip7 upregulation and GSK3 dephosphorylation in primary *Cln6* cells. As Zn^II^(atsm) is membrane-permeable, delivery of Zn to intracellular compartments may bypass impaired cellular Zn trafficking pathways. Labile Zn accumulation in primary *Cln6* neurons may be restricted to specific subcellular compartments, as previously reported for *Cln6* mouse brains [15], and may therefore not stimulate MT upregulation prior to addition of Zn^II^(atsm). Thus, Zn^II^(atsm) is likely to exert protective effects via a dual mechanism involving combined induction of Zip7 and MT. Zip7 induced by Zn^II^(atsm) may protect cells from toxic accumulation of compartmentalized labile metals by transporting these metals from the ER or Golgi, resulting in normalization of subcellular metal levels. This would promote an increase in bioavailable metals resulting in enhanced activity of Zn-requiring enzymes such as ALP (Figure 6D). Concomitantly, induction of MT is likely to result in MT-dependent sequestration of any excess metals that are liberated from the ER or Golgi in *Cln6* cells that are not directly required for enzymatic functions.

## Conclusions

Impaired metal homeostasis is a key hallmark of neurodegenerative disease. Here we show that aberrant biometal functions in CLN6 disease are driven by loss of the metal transporter, Zip7. The protective and metal modulating effects of Zn^II^(atsm) treatment *in vitro,* coupled with the proven success of metal btsc compounds in improving motor and cognitive functions in neurodegeneration models *in vivo*[37-39], suggest that Zn^II^(atsm) may be a candidate for NCL therapeutic trials.

## Abbreviations

ALP: Alkaline phosphatase; CNS: Central nervous system; ER: Endoplasmic reticulum; ICP-MS: Inductively coupled plasma mass-spectrometry; LSD: lysosomal storage disease; MT: Metallothionein; NCL: Neuronal ceroid lipofuscinosis.

## Competing interests

Patent protection has previously been sought by the University of Melbourne for the use of bis(thiosemicarbazones) for treatment of diseases. ARW and PSD are co-inventors on this patent application PCT/AU2007/001792, which is the subject of a commercialization contract between the University and a private company. The company has not funded nor contributed to research described in this manuscript.

## Authors’ contributions

AG, ARW and KMK designed research. AG, KMK, CD, JT, SJP, AC, GEL, JM, LB, IV, DM and JRL performed research. AG, IV, ARW and KMK analyzed the data. AG and KMK wrote the paper. ARW, PJC, JRL and JK provided critical revisions of the manuscript. IT, MH, PSD, JLH and SL synthesized reagents and collected sheep tissue samples. All authors read and approved the final manuscript.

## Supplementary Material

Additional file 1 **Zn**^**II**^**(atsm) treatment reduces aberrant neurite branching in primary *Cln6* cortical neurons.** (A) Neurite branching in control (black bars) and *Cln6* (white bars) primary cortical neurons treated for 1 h with Zn^II^(atsm) was determined by tubulin immunofluorescence. (B) Representative image of tubulin staining of primary cortical neurons. Images (>1,000 cells/well) were taken using the ArrayScan High Content Platform. (C) Analysis was performed using Neuronal Profiling software. Processed images show nuclei (blue), cell bodies (cyan), neurites (green or magenta for neurites from neighboring neurons for easier identification), branch points (yellow). Click here for file

Additional file 2 **Manganese and cobalt concentrations in the tissues of preclinical *CLN6* Merino sheep.** Click here for file

Additional file 3 **Zip7 concentrations are significantly and progressively reduced in *CLN6* sheep.** Immunoblots of homogenates (5 μg) isolated from the occipital lobe of 3 and 14 month-old control (C) or *CLN6* affected sheep (N = 3-4 per group) probed with antibodies directed against Zip7. Total ERK was used as a loading control. Click here for file

Additional file 4 **Unaltered metal transporter proteins in Merino *CLN6* affected sheep.** (A-C) Densitometry and representative immunoblots of homogenates (5–40 μg) isolated from the occipital lobe of 3, 7 and 14 month old control or *CLN6* affected Merino sheep (N = 3 per group) probed with antibodies directed against ZnT1 (A), ZnT3 (B) or ZnT6 (C). GAPDH, β-tubulin, total Akt or total ERK, as appropriate, were used as loading controls. Quantification was performed in ImageJ and metal transporter levels are expressed relative to those in control sheep at each age. C, control. Click here for file

Additional file 5 **Alterations to metal transporter protein concentrations in South Hampshire *CLN6* affected sheep.** Densitometry of western blots of homogenates (5–40 μg) isolated from the occipital lobe 12–14 month old control or *CLN6* affected South Hampshire sheep or *CLN5* heterozygote Borderdale sheep probed with antibodies directed against a range of metal transporters. GAPDH was used as a loading control. Quantification was performed in ImageJ and metal transporter levels are expressed relative to those in control sheep at each age. * *p <* 0.05, ** *p* < 0.01 by Student’s *t* test. C, control; H, heterozygote; A, affected. Click here for file

Additional file 6 **Zip7 loss is region specific in Merino *CLN6* affected sheep.** Densitometry analyses and representative western blots of Zip7 levels in the frontal lobe (A), parietal lobe (B) thalamus (C), cerebellum (D), brainstem (E), liver (F) and muscle (G) of 3, 7, and 14-month-old control and *CLN6* Merino sheep. GAPDH was used as a loading control. Quantitation was performed in ImageJ and metal transporter concentrations are expressed relative to those in control sheep at each age. ** *p* < 0.01, *** *p* < 0.001 by Student’s *t* test. Click here for file

Additional file 7 **Zip7 loss is region specific South Hampshire *CLN6 *** **affected sheep.** Densitometry of Zip7 western blots in the frontal lobe (A), thalamus (B), cerebellum (C), brainstem (D) in 12–14 month old control or *CLN6* affected South Hampshire sheep (N = 3 per group) or *CLN5* heterozygote Borderdale sheep (N = 2). GAPDH was used as a loading control. Quantification was performed in ImageJ and metal transporter concentrations are expressed relative to those in control sheep at each age. * *p <* 0.05, ** *p* < 0.01 by Student’s *t* test. C, control; H, heterozygote; A, affected. Click here for file

Additional file 8 **Zip7 co-localizes with ER in primary cortical neurons.** Primary mouse cortical neurons were fixed and stained with goat primary anti-Zip7 and rabbit primary anti-calnexin antibodies. Anti-rabbit AlexaFluor-568 and anti-goat AlexaFluor 488 dye labeled secondary antibodies were used to reveal Zip7 and calnexin expression. Nuclei were stained with DAPI. (A) DAPI, (B) Zip7 and (C) calnexin expression in primary cortical neurons was visualized by confocal microscopy using the Zeiss Meta confocal scanning laser microscope using a magnification of 20×. (D) Overlay images. Scale bars correspond to 20 μm. Click here for file
