# Supplementary figures and images for: Deregulation of subcellular biometal homeostasis through loss of the metal transporter, Zip7, in a childhood neurodegenerative disorder
Source: Acta Neuropathol Commun. 2014 Feb 28;2:25. doi: 10.1186/2051-5960-2-25 (PMC4029264; doi:10.1186/2051-5960-2-25)

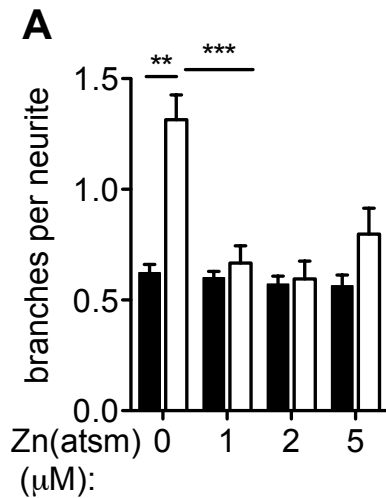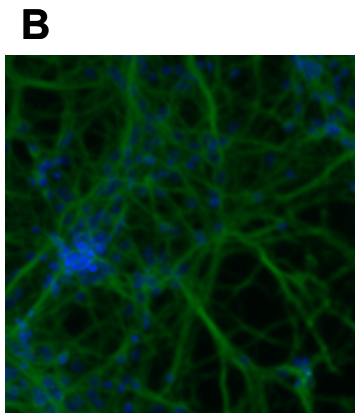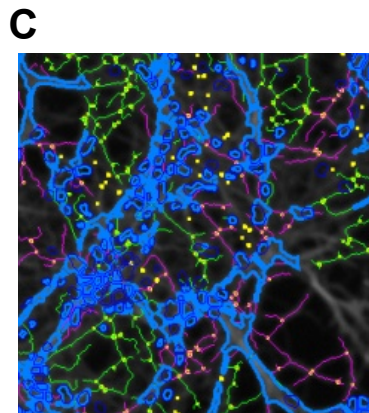

Supplement: Additional file 1 — Zn II (atsm) treatment reduces aberrant neurite branching in primary Cln6 cortical neurons. (A) Neurite branching in control (black bars) and Cln6 (white bars) primary cortical neurons treated for 1 h with ZnII(atsm) was determined by tubulin immunofluorescence. (B) Representative image of tubulin staining of primary cortical neurons. Images (>1,000 cells/well) were taken using the ArrayScan High Content Platform. (C) Analysis was performed using Neuronal Profiling software. Processed images show nuclei (blue), cell bodies (cyan), neurites (green or magenta for neurites from neighboring neurons for easier identification), branch points (yellow). [file 2051-5960-2-25-S1.PDF]

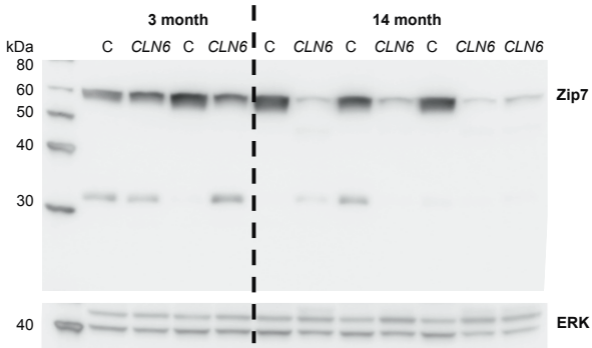

Supplement: Additional file 3 — Zip7 concentrations are significantly and progressively reduced in CLN6 sheep. Immunoblots of homogenates (5 μg) isolated from the occipital lobe of 3 and 14 month-old control (C) or CLN6 affected sheep (N = 3-4 per group) probed with antibodies directed against Zip7. Total ERK was used as a loading control. [file 2051-5960-2-25-S3.PDF]

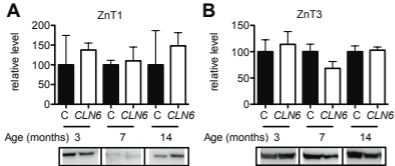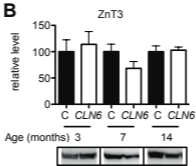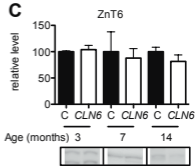

Supplement: Additional file 4 — Unaltered metal transporter proteins in Merino CLN6 affected sheep. (A-C) Densitometry and representative immunoblots of homogenates (5–40 μg) isolated from the occipital lobe of 3, 7 and 14 month old control or CLN6 affected Merino sheep (N = 3 per group) probed with antibodies directed against ZnT1 (A), ZnT3 (B) or ZnT6 (C). GAPDH, β-tubulin, total Akt or total ERK, as appropriate, were used as loading controls. Quantification was performed in ImageJ and metal transporter levels are expressed relative to those in control sheep at each age. C, control. [file 2051-5960-2-25-S4.PDF]

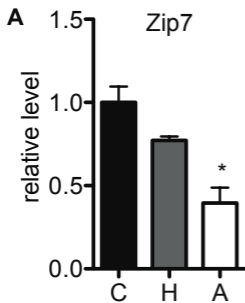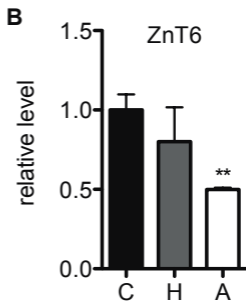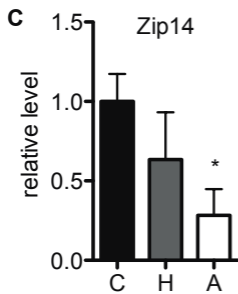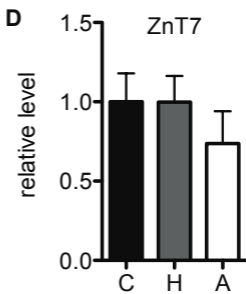

Supplement: Additional file 5 — Alterations to metal transporter protein concentrations in South Hampshire CLN6 affected sheep. Densitometry of western blots of homogenates (5–40 μg) isolated from the occipital lobe 12–14 month old control or CLN6 affected South Hampshire sheep or CLN5 heterozygote Borderdale sheep probed with antibodies directed against a range of metal transporters. GAPDH was used as a loading control. Quantification was performed in ImageJ and metal transporter levels are expressed relative to those in control sheep at each age. * p < 0.05, ** p < 0.01 by Student’s t test. C, control; H, heterozygote; A, affected. [file 2051-5960-2-25-S5.PDF]

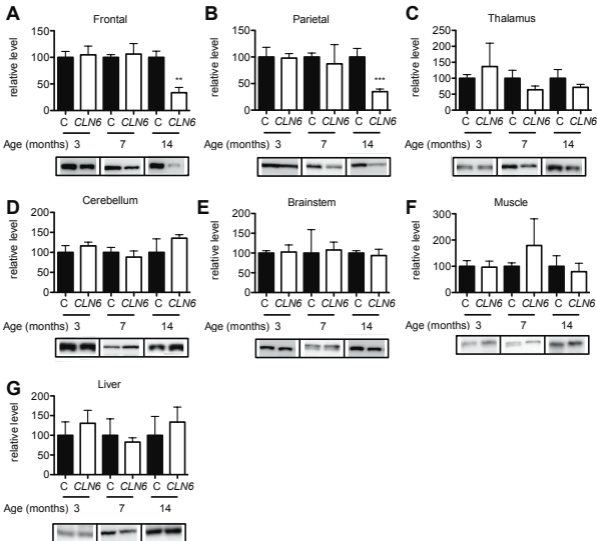

Supplement: Additional file 6 — Zip7 loss is region specific in Merino CLN6 affected sheep. Densitometry analyses and representative western blots of Zip7 levels in the frontal lobe (A), parietal lobe (B) thalamus (C), cerebellum (D), brainstem (E), liver (F) and muscle (G) of 3, 7, and 14-month-old control and CLN6 Merino sheep. GAPDH was used as a loading control. Quantitation was performed in ImageJ and metal transporter concentrations are expressed relative to those in control sheep at each age. ** p < 0.01, *** p < 0.001 by Student’s t test. [file 2051-5960-2-25-S6.PDF]

**A**

Frontal

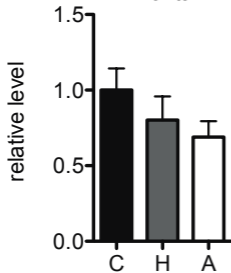**B**

Thalamus

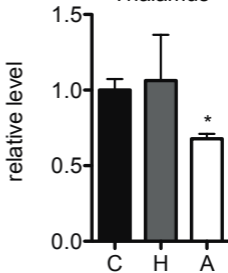**C**

Cerebellum

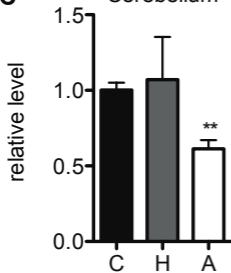**D**

Brainstem

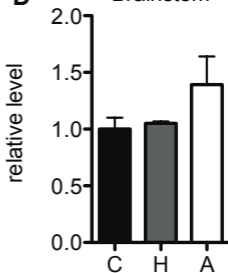

Supplement: Additional file 7 — Zip7 loss is region specific South Hampshire CLN6 affected sheep. Densitometry of Zip7 western blots in the frontal lobe (A), thalamus (B), cerebellum (C), brainstem (D) in 12–14 month old control or CLN6 affected South Hampshire sheep (N = 3 per group) or CLN5 heterozygote Borderdale sheep (N = 2). GAPDH was used as a loading control. Quantification was performed in ImageJ and metal transporter concentrations are expressed relative to those in control sheep at each age. * p < 0.05, ** p < 0.01 by Student’s t test. C, control; H, heterozygote; A, affected. [file 2051-5960-2-25-S7.PDF]

**A**

DAPI

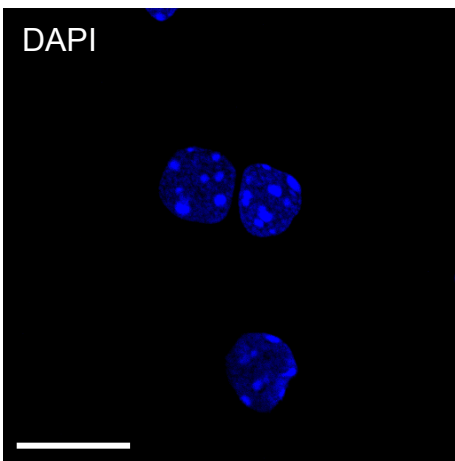**B**

Zip7

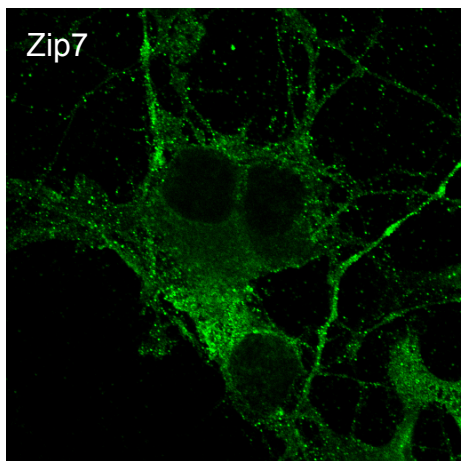**C**

Calnexin

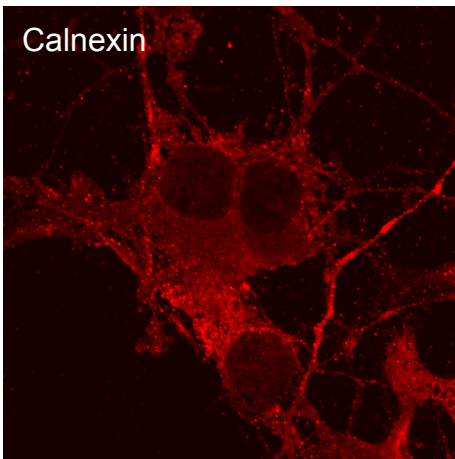**D**

Merge

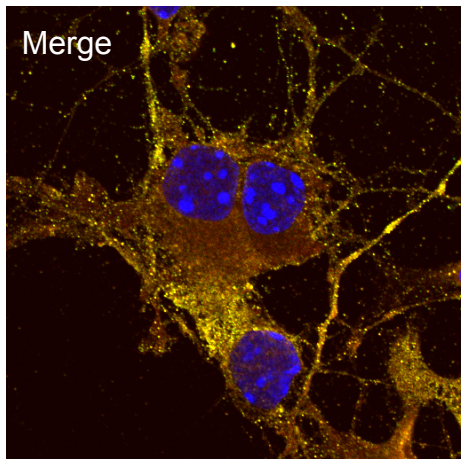

Supplement: Additional file 8 — Zip7 co-localizes with ER in primary cortical neurons. Primary mouse cortical neurons were fixed and stained with goat primary anti-Zip7 and rabbit primary anti-calnexin antibodies. Anti-rabbit AlexaFluor-568 and anti-goat AlexaFluor 488 dye labeled secondary antibodies were used to reveal Zip7 and calnexin expression. Nuclei were stained with DAPI. (A) DAPI, (B) Zip7 and (C) calnexin expression in primary cortical neurons was visualized by confocal microscopy using the Zeiss Meta confocal scanning laser microscope using a magnification of 20×. (D) Overlay images. Scale bars correspond to 20 μm. [file 2051-5960-2-25-S8.PDF]
