# Supplementary material for: Deregulation of subcellular biometal homeostasis through loss of the metal transporter, Zip7, in a childhood neurodegenerative disorder
Source: Acta Neuropathol Commun. 2014 Feb 28;2:25. doi: 10.1186/2051-5960-2-25 (PMC4029264; doi:10.1186/2051-5960-2-25)
Supplement: Additional file 2 — Manganese and cobalt concentrations in the tissues of preclinical CLN6 Merino sheep. [file 2051-5960-2-25-S2.DOCX]

**Additional file 2. Biometal concentrations in the tissues of preclinical *CLN6* Merino sheep.**

|  |  | Mn^#^ | | Co^#^ | |
| --- | --- | --- | --- | --- | --- |
|  | **Age** | **control** | ***CLN6*** | **control** | ***CLN6*** |
| Frontal lobe | **3** | 0.295±0.010 | 0.304±0.017 | 0.0072±0.0007 | 0.0079±0.0009 |
|  | **7** | 0.263±0.013 | 0.289±0.050 | 0.0092±0.0003 | 0.0087±0.0012 |
| Occipital lobe | **3** | 0.288±0.010 | 0.267±0.029 | 0.0069±0.0007 | 0.0075±0.0007 |
|  | **7** | 0.272±0.014 | 0.302±0.038 | 0.0094±0.0009 | 0.0103±0.0016 |
| Parietal lobe | **3** | 0.308±0.019 | 0.302±0.008 | 0.0068±0.0008 | 0.0078±0.0010 |
|  | **7** | 0.224±0.022 | 0.227±0.031 | ND | ND |
| Thalamus | **3** | 0.369±0.084 | 0.361±0.066 | 0.0052±0.0009 | 0.0057±0.0008 |
|  | **7** | 0.279±0.040 | 0.255±0.050 | ND | ND |
| Cerebellum | **3** | 0.413±0.023 | 0.443±0.051 | 0.0059±0.0007 | 0.0054±0.0010 |
|  | **7** | 0.297±0.037 | 0.273±0.035 | ND | ND |
| Brainstem | **3** | 0.422±0.035 | 0.422±0.025 | 0.0064±0.0007 | 0.0069±0.0008 |
|  | **7** | 0.291±0.034 | 0.266±0.018 | 0.0069±0.0007 | 0.0061±0.0006 |
| Liver | **3** | 3.609±0.575 | 2.848±0.257 * | 0.0696±0.0122 | 0.0527±0.0143 |
|  | **7** | 1.493±0.029 | 1.456±0.187 | 0.0378±0.0025 | 0.0393±0.0070 |
| Muscle | **3** | 0.102±0.022 | 0.136±0.049 | 0.0024±0.0004 | 0.0027±0.0010 |
|  | **7** | 0.156±0.084 | 0.134±0.046 | 0.0044±0.0012 | 0.0034±0.0008 |
| Plasma | **3** | 0.073±0.011 | 0.072±0.012 | 0.1075±0.0346 | 0.0972±0.0466 |
|  | **7** | 0.054±0.016 | 0.034±0.015 | 0.0129±0.0030 | 0.0082±0.0014 * |

* *p<*0.05, ** *p*<0.01, *** *p*<0.001 by Student’s *t* test.

^#^Metal concentrations in the CNS and peripheral tissues of 3 and 7 month old control and *CLN6* Merino sheep were measured using ICP-MS. The concentrations of manganese and cobalt in each tissue are expressed as mean ± S.D. Values correspond to μg metal/g tissue
